# Characterizing the personality and gray matter volume of chimpanzees that exhibit autism-related socio-communicative phenotypes

**DOI:** 10.1017/pen.2023.8

**Published:** 2023-10-27

**Authors:** William D. Hopkins, Michele Mulholland, Robert D. Latzman

**Affiliations:** 1 The University of Texas MD Anderson Cancer Center, Bastrop, TX 78602, USA; 2 Takeda Pharmaceuticals, Cambridge, MA, USA

**Keywords:** Chimpanzees, Personality, Social Cognition

## Abstract

Autism spectrum disorder (ASD) is a developmental disorder characterized by stereotypies or repetitive behaviors and impairments in social behavior and socio-communicative skills. One hallmark phenotype of ASD is poor joint attention skills compared to neurotypical controls. In addition, individuals with ASD have lower scores on several of the Big 5 personality dimensions, including Extraversion. Here, we examine these traits in a nonhuman primate model (chimpanzees; *Pan troglodytes*) to further understand the relationship between personality and joint attention skills, as well as the genetic and neural systems that contribute to these phenotypes. We used archival data including receptive joint attention (RJA) performance, personality based on caretaker ratings, and magnetic resonance images from 189 chimpanzees. We found that, like humans, chimpanzees who performed worse on the RJA task had lower Extraversion scores. We also found that joint attention skills and several personality dimensions, including Extraversion, were significantly heritable. There was also a borderline significant genetic correlation between RJA and Extraversion. A conjunction analysis examining gray matter volume showed that there were five main brain regions associated with both higher levels of Extraversion and social cognition. These regions included the right posterior middle and superior temporal gyrus, bilateral inferior frontal gyrus, left inferior frontal sulcus, and left superior frontal sulcus, all regions within the social brain network. Altogether, these findings provide further evidence that chimpanzees serve as an excellent model for understanding the mechanisms underlying social impairment related to ASD. Future research should further examine the relationship between social cognition, personality, genetics, and neuroanatomy and function in nonhuman primate models.

## ASD in Humans

Autism spectrum disorder (ASD) is a pervasive developmental disorder that can be viewed as an extreme of a set of personality traits. According to the DSM-5 (Association, 2013), ASD is characterized by impairments in three broad behavioral categories or phenotypes, including (1) stereotyped or repetitive behaviors and (2) impairments in social behavior, and (3) socio-communicative deficits, particularly early in development (Lord & Spence, 2006; Losh et al., 2009). DSM-5-TR combined the last two into a single socio-communicative domain, with “persistent impairment in reciprocal social communication and social interaction” as an essential feature of the disorder (Association, 2022).

The primary assessment of this core socio-communicative impairment has been measures of joint attention. Joint attention (JA), or joint engagement, refers to the dyadic process in which preverbal individuals begin to respond to (receptive joint attention, RJA), and initiate (initiating joint attention; IJA), nonverbal bids of communication via the use of gaze, gesture, and vocalizations (Adamson, 1996). Typically developing children progress through RJA and then IJA skills with a robust literature demonstrating that performance in these early JA abilities is predictive of language abilities at later points development (Butterworth, 1991; Baldwin, 1995; Carpenter, Nagell, Tomasello, Butterworth & Moore, 1998; Slaughter & McConnell, 2003; Nichols, Fox & Mundy, 2005; Mundy et al., 2007; Bottema-Beutel, 2016). With respect to ASD, several studies have shown that children with or at risk for the development of ASD are less inclined to engage in or appropriately develop JA skills compared to neurotypical controls (Mundy, Sigman, Ungerer & Sherman, 1986; Carpenter, Pennington & Rogers, 2002; Dawson et al., 2002; Osterling, Dawson & Munson, 2002; Dawson et al., 2004; Landa, Holman & Garrett-Mayer, 2007; Sullivan et al., 2007; Wetherby, Watt, Morgan & Shumway, 2007; Landa, 2008; Adamson, Bakeman, Deckner & Romski, 2009).

Individuals with a diagnosis of, or at risk for, ASD have also been reported to differ in personality including among the Big 5 personality traits. For instance, in a recent meta-analysis, Lodi-Smith, Rodgers, Cunningham, Lopata and Thomeer (2019) reported that individuals with increasing ASD characteristics (based on various ASD scales) had lower Big 5 scores, particularly in Extraversion, Openness, Conscientiousness, Agreeableness and Emotional Stability. Similarly, group comparisons found that individuals with ASD scored lower on these Big 5 traits compared to neurotypical control groups. Here, we argue for similar linkages in chimpanzees (Pan troglodytes) and related these to local variations in gray matter volume.

## Joint attention and personality in nonhuman primates

Nonhuman animals are important models for studies on the genomic and neural systems that contribute to behaviors that are used as model phenotypes of ASD (Bauman & Schumann, 2018; Silverman et al., 2022). In nearly all primate species used as animal models for ASD, different dimensions of social behavior or personality have been the behavioral phenotypes of interest (Yirmiya et al., 2006; Mahovetz, Young & Hopkins, 2016; Proctor, Calcutt, Burke & de Waal, 2016; Wilson et al., 2017; Parker et al., 2018; Weiss, Wilson & Hopkins, 2021; Gunter et al., 2022). By contrast, however, there are surprisingly few studies that have focused on overt measures of social cognition, including joint attention (Hopkins et al., 2014a). This is unfortunate because JA abilities are not uniquely human but have been reported extensively in all great ape and, to a lesser extent, in more distantly related primate species (e.g., rhesus macaques, capuchins, and marmosets; Clark, Elsherif & Leavens, 2019). For instance, chimpanzees and other great apes will follow gaze and pointing gestures to objects and will return objects that are requested from them based on vocal and gestural cues. Chimpanzees and other great apes will also gesture to foods or objects that are otherwise out of their reach while alternating their gaze between the referent and a human experimenter, though there is some debate regarding nonhuman primates to engage in declarative pointing (Krause, Udell, Leavens & Skopos, 2018; Lyn, Greenfield, Savage-Rumbaugh, Gilliespie-Lynch & Hopkins, 2011; Tomasello, 2008).

Many studies have shown that chimpanzees and other nonhuman primates show different dimensions of personality based on subjective ratings provided by humans who frequently interact with or observe the subjects (Gosling, 2001; Freeman & Gosling, 2010; Weiss, King & Murray, 2011; Freeman et al., 2013; Staes et al., 2015; Staes et al., 2016). Using human caretaker ratings, the literature has generally converged on four (Dominance, Extraversion, Conscientiousness, and Agreeableness) to six factors (Dominance, Extraversion, Conscientiousness, Agreeableness, Neuroticism, and Openness) of personality traits among chimpanzees including traits such as dominance, neuroticism, Extraversion, agreeableness, and others (King & Figueredo, 1997; Latzman, Hopkins, Keebaugh & Young, 2014).

## Current study

In this paper, we evaluated whether chimpanzees might serve as an important model species for understanding the neurobiology of ASD. First, we analyzed archival social cognition and personality data in a sample of 189 chimpanzees to explore the notion that certain chimpanzees may exhibit consistency in these two ASD-like phenotypes. Specifically, we examined whether chimpanzees that perform poorly on measures of joint attention also vary in their personality and, in particular, those personality dimensions that reflect sociality in a broad sense of the term including both behavior and communication. Second, we examined whether common neuroanatomical substrates were associated with individual variation in joint attention and measures of personality in a subsample of 155 chimpanzees for which structural magnetic resonance image (sMRI) scans were available. For the purposes of the current study, we investigated associations between performance on two measures of RJA and the 5 personality dimensions described in Latzman et al. (2014) in a sample of 189 chimpanzees.

## Methods

### Subjects

For the behavioral analyses of the association between joint attention and personality, we used archival data from 189 chimpanzees who were housed at The University of Texas MD Anderson Cancer Center (*N* = 108) and Emory (previously Yerkes) National Primate Research Center (*N* = 81). Subject information including sex and rearing history of the chimpanzees in this study can be found in Table 1. Notably, beginning in the 1980s, the National Institutes of Health funded captive chimpanzee breeding programs that were designed to increase the available apes for use in biomedical and behavioral research. Many of the females in the breeding program successfully birthed and subsequently cared for their new offspring (herein mother-reared, MR, *n* = 107); however, some females engaged in poor or inadequate maternal care that required an intervention to save the infant’s life. These newborn chimpanzees were raised in a human nursery-setting with same age peers until ∼3 years of age at which point they were integrated into larger mixed-age and sex groups (herein nursery-reared, NR, *n* = 47). It has been well documented that standard nursery-rearing of chimpanzees and other nonhuman primates can result in poor species-specific social behavioral development, differences in personality as well as induced stereotypies (Sackett, Ruppenthal & Elias, 2006; Zhang, 2017), and characteristics observed in individuals with a diagnosis of ASD. For this reason, we were specifically interested in the possible independent or interactive effect of rearing history and joint attention abilities on the personality measures. Beside the MR and NR apes, there was also a third rearing group of chimpanzees that were wild-born (herein WB, *n* = 35). WB chimpanzees were brought to the USA from Africa prior to the 1974 CITES (Convention on International Trade in Endangered Species) ban on their importation. They were mostly the oldest chimpanzees in the sample and were presumably mother-reared; but because we did not know their exact rearing histories, we considered them as a distinct group in analyses.

**Table 1.** Subject distribution for each analysis reported here

	Rearing group		
	MR	NR	WB
Behavioral analysis (*n* = 189)			
Males	43	23	10
Females	64	24	25
Total	107	47	35
Neuroimaging analysis (*n* = 155)			
Males	38	23	6
Females	47	23	18
Total	85	46	25

### Personality measures

Using data from a 43-item personality questionnaire originally developed by King and Figueredo (1997) with ratings by caregivers of more than 200 chimpanzees from two separate cohorts, Latzman et al. (2014) used a hierarchical structural analysis and settled on a five-factor structure solution at the most differentiated level of the hierarchy. This structure included the personality dimensions of (low) Conscientiousness, Dominance, Extraversion, Agreeableness, and (low) Intellect. We used this archival personality data in the current study (Latzman et al., 2014), and the items from the questionnaire that loaded on each factor structure are shown in Table 2.

**Table 2.** Varimax rotated exploratory factor analysis of chimpanzee personality questionnaire: five-factor solution

	Factor				
Item	1 (low C)	2 (Dom)	3 (E)	4 (A)	5 (low I)
Excitable	**0.84**	−0.06	0.07	−0.01	−0.03
Impulsive	**0.77**	0.13	0.13	−0.09	0.05
Irritable	**0.75**	0.21	−0.3	−0.19	0.05
Erratic	**0.74**	0.06	−0.19	−0.13	0.33
Stable	−0.**71**	0.19	−0.1	0.38	0.00
Aggressive	**0.65**	**0.54**	0.08	−0.16	0.08
Defiant	**0.65**	0.44	0.03	0.03	−0.01
Reckless	**0.65**	0.42	0.23	−0.23	0.08
Jealous	**0.58**	0.35	0.17	0.01	0.01
Gentle	−0.**55**	−0.4	0.01	0.49	−0.03
Stingy	**0.54**	**0.5**	−0.1	−0.05	−0.01
Predictable	−0.47	0.15	−0.08	0.32	−0.02
Autistic	0.47	−0.22	−0.32	0.18	0.13
Submissive	−0.12	−0.**84**	−0.14	0.05	0.12
Dominant	0.33	**0.81**	0.04	0.09	0.05
Dependent	−0.02	−0.**78**	0.05	0.12	0.22
Independent	0.06	**0.73**	−0.11	0.01	−0.17
Fearful	0.21	−0.**72**	−0.05	0.10	0.00
Timid	0.04	−0.**66**	−0.38	0.10	0.38
Cautious	−0.26	−0.**63**	−0.17	0.35	0.01
Bullying	0.55	**0.63**	0.09	−0.14	0.01
Decisiveness	0.15	**0.55**	−0.07	0.12	−0.47
Persistence	0.46	0.48	0.26	0.12	−0.08
Manipulative	0.41	0.46	0.05	0.36	0.07
Playful	0.05	−0.02	**0.88**	0.12	−0.01
Active	0.31	0	**0.82**	−0.09	0.08
Solitary	0.11	−0.07	−0.**81**	−0.09	0.12
Lazy	−0.22	0.02	−0.**81**	0.09	0.13
Depressed	0.23	−0.22	−0.**72**	−0.05	0.28
Inquisitive	0.08	0.04	**0.72**	**0.22**	−0.27
Sociable	−0.28	0.06	**0.62**	0.49	−0.08
Inventive	0.17	0.2	0.38	0.29	−0.22
Protective	−0.04	0.09	0.02	**0.8**	0.04
Helpful	−0.12	−0.12	0.08	**0.71**	−0.16
Sympathetic	−0.43	−0.25	0.08	**0.65**	−0.06
Sensitive	−0.22	−0.34	0.14	**0.52**	−0.43
Affectionate	−0.4	−0.12	0.46	**0.51**	−0.19
Friendly	−0.45	−0.18	0.42	**0.5**	−0.03
Imitative	0.19	−0.33	0.25	0.37	0.19
Disorganized	0.31	−0.18	−0.23	−0.12	**0.72**
Intelligent	0.02	0.21	0.17	0.36	−0.**69**
Clumsy	0.25	−0.18	−0.31	0.16	**0.68**
Unemotional	−0.39	−0.07	−0.38	0.07	0.49

*n* = 174. Low C = low Conscientiousness. Dom = Dominance. E = Extraversion. A = Agreeableness. Low I = low Intellect. Loadings ≥ 0.50 are in **boldface**. From Latzman et al. (2014).

### Behavioral measures

For many of the chimpanzees rated for personality in the Latzman et al. (2014) paper, we also had measures of RJA using the same methods and tasks as those used by researchers working with children with and without ASD. Notably, we used data collected from two joint attention tasks, referred to as the MUNDY and DAWSON tasks. The methods used for their measurement have been described in detail elsewhere (Hopkins & Latzman, 2021) (see Table 1 for sample sizes).

Briefly, the MUNDY task was designed to model those used in a previous study of human children (Mundy et al., 2007). Each chimpanzee received 24 test trials, divided over 4 sessions, with only one 6-trial session performed per day. Prior to beginning the task, the experimenter placed two PVC (poly-vinyl-chloride) stations as high and far laterally apart on the cage mesh as possible, but within 1–2 meters of the focal subject. The experimenter positioned themselves in front of the subject an equal distance between the two PVC stations and engaged them in some basic husbandry training tasks. While the subject was actively engaged with the experimenter, the experimenter stopped interacting with the subject and pointed (full arm extended and maintained throughout the trial) and looked toward one of the PVC stations (the cued PVC) and said the chimpanzee’s name with increasing emphasis. If the subject looked at, oriented toward, or touched the cued PVC station during this time, they received a “1”, indicating a correct response. If the subject did not look at, orient toward, or touch the *cued* PVC, or if they instead looked at, oriented toward, or touched the *non-cued* PVC pipe, then they received a score of “0” for that trial, indicating an incorrect response. This process was repeated for all six trials within a session, with each trial separated by the experimenter re-engaging the subject with the basic husbandry training task. The experimenter randomly alternated which of the PVC stations was the cued stimulus. The dependent measure was the proportion of correct responses across the 24 trials.

For the DAWSON task (Dawson et al., 2004), at the onset of each trial, a human experimenter would engage in basic husbandry training activities with the focal subject. When the experimenter sensed that the focal chimpanzee was engaged and facing them, they would stop their action and initially look over the shoulder of the subject for 5 s, as if there were an object behind them. At the end of this cue, the chimpanzee’s behavior was recorded for 15 s. If they looked behind them, they were given a score of 4 and the trial ended. If the focal chimpanzee subject did not look behind them, the experimenter re-engaged the subject in husbandry training behavior. When the experimenter judged the subject to be engaged and facing them, they stopped and again looked over the focal subject’s shoulder and pointed as if there were an object behind the ape. Following this cue, the chimpanzee was again observed for 15 s, and if they looked behind them, they were given a score of 3 and the trial ended. As before, if the chimpanzee did not look behind them, the experimenter re-engaged the chimpanzee in husbandry training behavior. When the experimenter again sensed that the chimpanzee was engaged, they stopped and now looked over the focal subject’s shoulder, pointed, and vocally prompted the chimpanzee to an object behind them. Following this cue, the chimpanzee’s response was recorded for 15 s and if they looked behind them, they were given a score of 1 and the trial ended. If the subject failed to look behind them at the end of this phase of the trial, they were given a score of 0. Each chimpanzee received four trials, and the trials were administered across different days. The outcome measure was the sum of their performance scores across the four trials and ranged between 0 and 16.

### MRI acquisition and processing

The method of MRI collection, post-image processing steps and voxel-based morphometry (VBM) have been described in previous studies (Mulholland et al., 2020). Briefly, sMRI scans, RJA and personality data were available in 155 chimpanzees. sMRI scans were collected on either a 1.5T (*n* = 93) or a 3T (*n* = 62) scanner from chimpanzees during their annual physical examinations. The sMRI scans were subsequently resampled at .625 mm isotropic resolution, aligned in the AC–PC axis, skull-stripped using the Brain Extraction Tool function in FSL (Smith, 2002; Jenkinson, Pechaud & Smith, 2005), N4 bias-corrected in 3DSlicer (www.3Dslicer.org) (Boyes et al., 2008; Tustison et al., 2010), and denoised using the MRI Denoising Package for MATLAB (R2015b; Mathworks, Natick, Massachusetts, USA) (Coupé et al., 2008). The sMRI preprocessed scans were then processed in the VBM pipeline within FSL (Functional MRI of the Brain Software Library; fsl.fmrib.ox.ac.uk/fsl/fslwiki/FSLVBM), which included segmentation, creation of a study-specific template and subsequent linear registration, followed by nonlinear registration of segmented gray matter volume to the study-specific gray matter template. The modulated gray matter volumes were then smoothed with an isotropic Gaussian kernel with a sigma of 2 mm.

### Data analyses

To create a composite score of joint attention based on performance for the two measures, the data for each task were converted to standardized *z*-scores and then averaged to create an overall RJA performance score (Mean_RJA). We then classified chimpanzees as performing above average (*z*-scores > 0, assigned value = 1) or below average (*z*-scores <= 0, assigned value = 0) based on their Mean_RJA value (JA + *n* = 114, JA-, *n* = 75). We ran a multivariate analysis of covariance (MANCOVA) with sex, rearing history, and JA classification as the between-group factors, the factor scores for the five personality dimensions as the outcome measures, and age as the covariate. Alpha was set to *p* < 0.05 (two-tailed), and any necessary *post hoc* tests were performed using Tukey’s HSD (honestly significant difference) test.

Rather than characterizing RJA performance as a composite score based on the average performance of both the MUNDY and DAWSON tasks, we also performed separate analyses for each task. For the MUNDY task, we computed binomial *z*-scores for each subject based on their performance on the 24 test trials to evaluate whether individual performance was significantly better than chance (50% correct). Subjects with a binomial *z*-score ≥ 1.64 were classified as passing (P) the test, while all others were classified as failing (F). To create a binary performance measure for the DAWSON task that was comparable to the outcome measures for the MUNDY task, we calculated the percentage of trials (out of 4) in which the chimpanzees scored either a 2, 3, or 4. There were too few trials on the DAWSON task to perform binomial *z*-scores. For this reason, the dependent measure for the DAWSON task was the percentage of trials in which they looked behind them (scored as correct; as a 1, 2, or 3) out of the four test trials. Chimpanzees that scored a 0 were classified as failing (F), while chimpanzees with scores of 1, 2, or 3 were classified as passing (P).

As in previous studies, we used the quantitative genetics program SOLAR (Sequential Oligogenetic Linkage Analysis Routines, 8.4.2) to estimate heritability for the personality dimensions and mean RJA scores (with age, sex, and rearing history as covariates) within this sample of chimpanzees based on their known pedigree. SOLAR uses a variance component approach to estimate the polygenic component of variance when considering the entire pedigree (Rogers et al., 2007; Fears et al., 2009; Kochunov et al., 2010). Narrow-sense heritability (h^2^) is the proportion of total phenotypic variance that is attributable to additive genetic effects. Total phenotypic variance attributable to genetic and nongenetic variables (e^2^; e.g., error variance and environmental effects) is constrained to a value of 1; thus, all nongenetic contributions to the phenotype are equal to 1 – h^2^.

## Results

### Associations between joint attention and personality ratings

**Figure 1.**
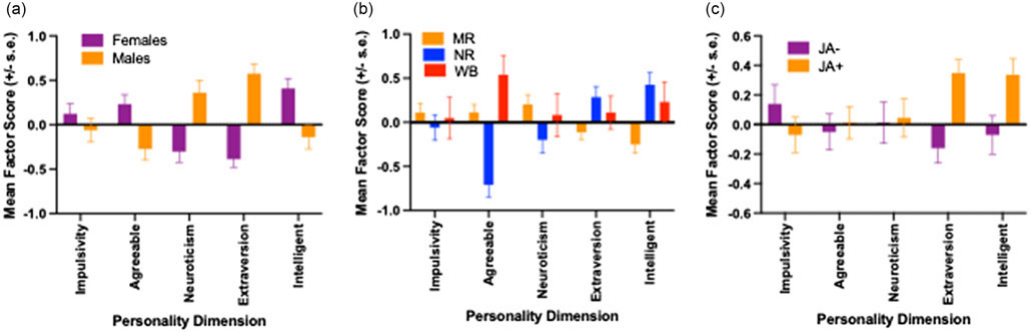
Mean factor score (+/- s.e.) for each personality dimension between (a) males and females, (b) different rearing groups, and (c) chimpanzees that performed above (JA+) or below (JA-) a standardized z-score of 0. MR = mother-reared, NR = nursery-reared, WB = wild-born.

The MANCOVA revealed significant main effects for sex [*F*(5, 172) = 20.21, *p* < 0.001, partial n^2^ = 0.370], rearing history [*F*(10, 346) = 5.84, *p* < 0.001, partial n^2^ = 0.145], and JA classification [*F*(5, 172) = 3.98, *p* < 0.002, partial n^2^ = 0.104]. The mean factor scores (+/- s.e.) for each personality dimension between sexes, rearing groups, and chimpanzees in the different JA classification groups are shown in Figures 1a–c. The univariate *F*-tests revealed that females higher Agreeableness [*F*(1, 176) = 9.92, *p* < 0.002, partial n^2^ = 0.053] and Intellect [*F*(1, 176) = 11.11, *p* < 0.001, partial n^2^ = 0.059] scores than males, whereas males had higher Dominance [*F*(1, 176 = 13.66, *p* < 0.001, partial n^2^ = 0.072] and Extraversion *[F*(1, 176) = 50.03, *p* < 0.001, partial n^2^ = 0.221] scores. For the main effect of rearing, the univariate *F*-tests indicated significant effects for Agreeableness [*F*(2, 176) = 16.95, *p* < 0.001, partial n^2^ = 0.161], Extraversion [*F*(2, 176) = 4.29, *p* = 0.015, partial n^2^ = 0.046] and Intellect [*F*(2, 176) = 8.63, *p* < 0.001, partial n^2^ = 0.089]. For Agreeableness, *post hoc* analysis using Bonferroni’s correction procedure revealed that NR chimpanzee had lower values than both MR and WB apes; however, MR and WB chimpanzees did not differ in Agreeableness who did not differ from each other. By contrast, for Extraversion and Intellect, NR chimpanzees had higher values than MR but not WB apes who did not differ significantly from each other. Lastly, for the JA classification variable, significant main effects were found for Extraversion [*F*(1, 176) = 13.64, *p* < 0.001, partial n^2^ = 0.072] and Intellect [*F*(1, 176) = 5.80, *p* = 0.017, partial n^2^ = 0.032]. For both personality dimensions, JA+ apes had higher values than JA individuals.

### Associations between personality ratings and each separate joint attention task

**Figure 2.**
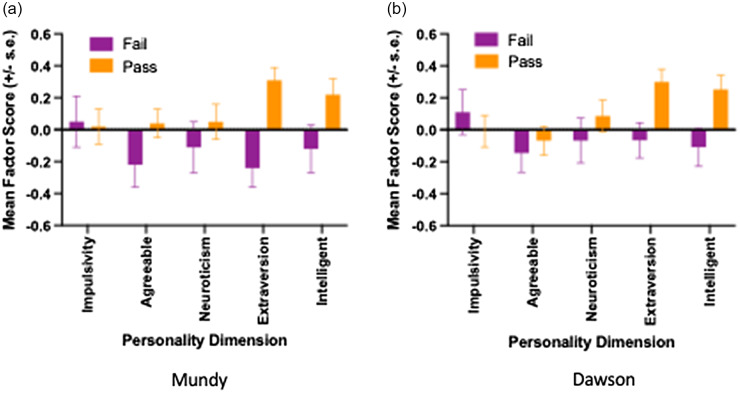
Mean factor score (+/- s.e.) for each personality dimension between chimpanzees that were judged to pass or fail the (a) MUNDY or (b) DAWSON receptive joint attention tasks.

Shown in Figures 2a and b are the mean factor scores (+/− s.e.) in chimpanzees that we judged to pass or fail the DAWSON and MUNDY tasks. Univariate *F*-tests revealed significant main effects of performance for the MUNDY [*F*(1, 176) = 14.39, *p* < 0.001, partial n^2^ = 0.076] and DAWSON [*F*(1, 197) = 6.97, *p* < 0.009, partial n^2^ = 0.034] tasks on the Extraversion personality scores with subjects that passed the tasks having higher Extraversion scores than those that failed. For the DAWSON task, we also found a significant effect of performance on Intellect personality scores [*F*(1, 197) = 5.19, *p* = 0.024, partial n^2^ = 0.026], chimpanzees that passed the DAWSON task had higher Intellect scores.

### Heritability and genetic correlates between joint attention and personality

Within this sample, and consistent with previous results reported by Latzman et al. (2015), we found that Extraversion, Agreeableness, and Dominance were significantly heritable while Intellect and Impulsivity were not (see Table 3). The mean RJA values were also found to be significantly heritable in this sample of chimpanzees. Interestingly, we found a borderline significant genetic correlation between the Mean RJA and Extraversion scores (*rhoG* = 0.678, se = 0.334, *p* = 0.06) but not with Agreeableness (*rhoG* = 0.416, se = 0.682, ns) nor Dominance (*rhoG* = −0.142, se = 0.337, ns). This suggests that common genes may underlie both RJA abilities and Extraversion scores in chimpanzees.

**Table 3.** Heritability in personality dimensions and mean RJA scores in 189 chimpanzees

Measure	*h* ^2^	s.e	*p*	Covariates
Impulsivity	0.021	0.154	0.498	None
Agreeableness	0.217	0.112	0.015	None
Dominance	0.269	0.108	0.001	None
Extraversion	0.234	0.124	0.008	Sex
Intellect	0.000	——	0.500	Rearing
MEAN_RJA	0.392	0.153	0.001	Age

h^2^ = additive genetic variance; s.e. = standard error.

### Neuroanatomical correlates of Extraversion and joint attention

The analyses of the behavioral data showed that chimpanzees who perform poorly on measures of RJA show lower Extraversion scores based on personality ratings. Further, these results were consistent across both measures of RJA. We next considered the neuroanatomical correlates of individual differences in the RJA/Extraversion scores with gray matter volume in the chimpanzees. For this set of analyses, we performed a VBM conjunction analysis from MRIs scans that were available in subsample of 155 chimpanzees within the original sample. The distribution of male and female MR, NR, and WB apes included in this analysis can be found in Table 1.

**Figure 3.**
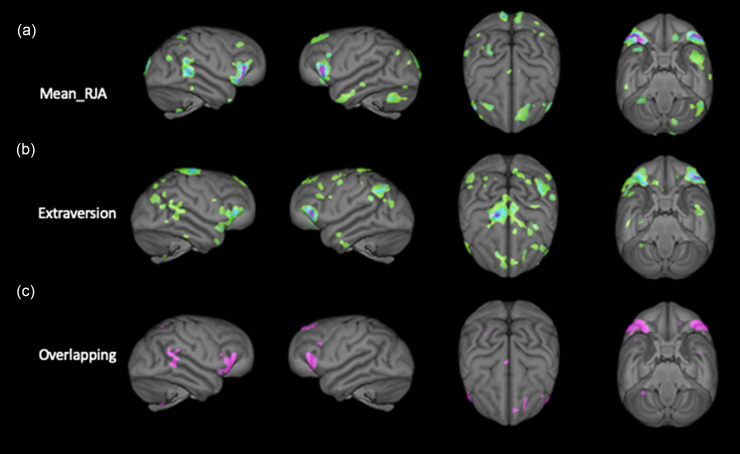
Gray matter regions (colored) that are correlated with (a) Mean_RJA scores, (b) Extraversion scores, and (c) brain regions that are overlapping and associated with both Mean RJA and Extraversion scores.

First, we performed two separate VBM analyses with alpha set to *p* < 0.01 (uncorrected) on the smoothed gray matter volumes. In the first analysis, we regressed the mean RJA scores on gray matter while controlling for sex, scanner magnet, and rearing history (associated brain areas are shown in Figure 3a). In the second analysis, we regressed the Extraversion factor scores on gray matter volume while controlling for sex, scanner magnet, and rearing history (associated brain areas are shown in Figure 3b). Next, we binarized the gray matter output volumes for each VBM analysis and combined them to create a single volume and then thresholded to show only those brain regions associated with both mean RJA performance and the Extraversion personality scores (overlapping areas are shown in Figure 3c). For the conjunction analysis, in total, five brain regions were overlapping between the two VBM analyses, including the right posterior middle and superior temporal gyrus, bilateral inferior frontal gyrus, left inferior frontal sulcus, and left superior frontal sulcus.

## Discussion

One finding from this study is that chimpanzees who exhibit poor performance on joint attention (RJA) as a measure of social cognition also show lower observer-rated Extraversion personality scores but not lower values on other personality dimensions, save Intellect. A second finding is that chimpanzees who perform poorly on measures of social cognition and have lower Extraversion scores also have lower gray matter volumes in several brain regions within the social brain network (e.g., middle and superior temporal gyrus, inferior frontal gyrus; Adolphs, 2009; Lewis, Rezaie, Brown, Roberts & Dunbar, 2011; Sliwa & Freiwald, 2017) and the posterior attentional network.

With respect to the findings of a positive association between RJA performance and Extraversion, the results were consistent with our hypothesis. Chimpanzees appear to exhibit a suite of social cognition and personality traits that reflect, in a broad sense, social impairments, a key phenotypic dimension of ASD and other neurodevelopmental disorders. These measures also fall well within the NIMH RDoC (Research Domain Criteria) systems for social processes constructs (Insel et al., 2010), reinforcing the view that chimpanzees are an excellent model species for studies on mechanisms that govern individual differences in social impairments, including ASD (Latzman & Hopkins, 2016). Though nonverbal behavior and communication were behavioral constructs included in the original RDoC social processes domain, there has been a recent call to more broadly include measures of social attention within this domain (Mundy, 2023). The findings reported support the argument that social attention should be included within the RDoC social process domain. Moreover, our findings further demonstrate the value of basic research with chimpanzees as a model species for understanding the neurobiological basis of typical and atypical psychological functions defined within the RDoC social processes domain and perhaps others.

We found significant rearing effects on the personality dimensions Agreeableness, Extraversion, and Intellect. In the case of Agreeableness, MR chimpanzees scored significantly higher than NR but not WB individuals. By contrast, NR chimpanzees had higher Extraversion and Intellect scores compared to MR but not WB individuals. Some have hypothesized that nursery-rearing induces ASD-like phenotypes in nonhuman primates (Nelson & Winslow, 2009; Bauman & Schumann, 2018) but, at face value, our findings do not entirely support this claim if we assume that the Extraversion dimension of personality best captures ASD-like dimensions. Recall that NR chimpanzees scored higher not lower than MR chimpanzees (see Figure 1b) on the Extraversion dimension. The MR chimpanzees did score higher on Agreeableness, and the items that loaded on this personality dimension of personality included the adjectives helpful, sympathetic, sensitive, affectionate, and friendly. Each of these items are indicative of prosocial and empathic tendencies and therefore may reflect another aspect of social impairment that may be less driven by social cognitive processes than those associated with Extraversion. Importantly, the conflicting results on the role of rearing on Agreeableness and Extraversion points to the limitations of interpreting personality data derived from subjective ratings in the context of their association with motivational, affective, and cognitive process that are manifest in observable behavior.

We also found that Extraversion, Agreeableness, and Dominance were significantly heritable as was performance on the joint attention tasks, which has been reported in our previous studies (Hopkins & Latzman, 2021; Hopkins et al., 2014b; Latzman, Freeman, Schpairo & Hopkins, 2015). Unique to this study was the borderline significant genetic correlation between mean RJA performance and Extraversion. This suggests that common but as yet unknown genes may underlie the expression of these two ASD-relevant traits. For instance, DeYoung (2010) has hypothesized that the personality trait Extraversion is derived, in part, from the behavioral approach system and mediated by genes that regulate dopaminergic neurotransmitter systems. Interestingly, Staes et al. (2022) recently reported that DNA methylation values for the dopamine receptor DRD2 were associated with Extraversion scores in chimpanzees. Performance data for the DAWSON RJA task were available in the 51 chimpanzees included in the Staes et al. report. Therefore, we tested whether the performance data on the DAWSON task was associated with DNA methylation factor scores for the DRD2 gene reported in the Staes et al. paper. We found that performance on the DAWSON task correlated positively with the first principal component DRD2 factor score (*r* = 0.461, df = 49, *p* < 0.001), the same component that was linked to the Extraversion scores reported by Staes et al. Thus, DNA methylation values for DRD2 derived from blood samples were associated with both Extraversion and joint attention scores in a sample of 51 chimpanzees.

The conjunction analysis showed that there were five main brain regions associated with both higher levels of Extraversion and social cognition. These regions included the right posterior middle and superior temporal gyrus, bilateral inferior frontal gyrus, left inferior frontal sulcus, and left superior frontal sulcus. Each of these brain regions are within the social brain network, and therefore it follows that their gray matter volume is associated with Extraversion and joint attention scores and are consistent with some previous findings (Hopkins et al., 2014b). Indeed, the inferior frontal gyrus of the chimpanzee brain is the homolog to the Pars opercularis in the human brain which is one of three morphological regions comprising Broca’s area (Keller, Roberts & Hopkins, 2009; Schenker et al., 2010), whereas the posterior temporal gyrus overlaps with Wernicke’s area (Spocter et al., 2010). In light of the role of the inferior frontal gyrus in communicative functions, their association with performance, particularly for the joint attention measures (which are impacted in ASD) is noteworthy. We would further add that analyses of gray matter covariation have found reduced gray matter volumes within the inferior frontal gyrus in ASD compared to controls (Mei et al., 2020). Moreover, the severity in the symptoms used to diagnose ASD which included the Autism Diagnostic Observation Scale (ADOS) and  Autism Diagnostic Interview (ADI) scales were also associated gray matter volume within the inferior frontal gyrus.

The current study is not without limitations. Notably, the joint attention, personality, and brain imaging data were collected over different time points in the chimpanzees’ lives, and ideally these would have been obtained on or about the same time point in their lifespan. In addition, for the separate analysis of performance on the MUNDY and DAWSON task on the personality dimensions, the classification of chimpanzees into the pass or fail groups was somewhat arbitrarily determined, particularly for the DAWSON task, rather than based on some psychometric or clinical cut point. That stated, while controlling for rearing history, sex, and age, we find significant positive association between the Extraversion scores and the raw MUNDY (*r* = 0.369, *p* < 0.001) and DAWSON (*r* = 0.194, *p* = 0.012) performance scores. Thus, the overall pattern of results is consistent independent of the approach in the analysis of the data. Finally, personality was measured based on ratings by humans familiar to the chimpanzees. Presumably, the different dimensions of personality found in chimpanzees (and other species) are manifest of specific behavioral dispositions and motivational states, but these are not always apparent or obvious (Corr, DeYoung & McNaughton, 2013). Arguably, testing for associations between objective and quantifiable traits might be more useful in terms of understanding the biological and neural basis of personality.

In summary, limitations notwithstanding, we found that chimpanzees with poorer JA performance had lower Extraversion scores, both Extraversion and JA performance are heritable, and that both phenotypes are related to lower gray matter volumes in several brain regions within the social brain network and the posterior attentional network. These findings provide further evidence that chimpanzees serve as an excellent model for understanding the mechanisms underlying social impairment, generally, and social impairment associated with psychiatric disorders such as ASD, more specifically. Future research should further examine other genetic and neural correlates of social impairment in chimpanzees using archival data and examine these relationships in other nonhuman primate models. In addition, alternative imaging technologies that quantify anatomical or functional connectivity may reveal more relevant results, particularly as it relates to networks of connected brain regions.
